# Pediatric tumor microenvironment: developmental dynamics and therapy resistance

**DOI:** 10.3389/fonc.2025.1663975

**Published:** 2025-11-10

**Authors:** Noreen Grace George, Bhavika Rishi, Himanshu Dhanda, Gratial Theres Joseph, Sandeep Kumar Swain, Manpreet Kaur, Neetu Kushwaha, Raj Kamal, Pranay Tanwar, Sufian Zaheer, Shamsuz Zaman, Amitabh Singh, Fouzia Siraj, Aroonima Misra

**Affiliations:** 1 Indian Council of Medical Research-National Institute of Child Health Development Research, New Delhi, India; 2 Manipal Academy of Higher Education (MAHE), Manipal, India; 3 All India Institute of Medical Sciences, New Delhi, India; 4 Vardhman Mahavir Medical college, Safdarjung Hospital, New Delhi, India; 5 Indian Council of Medical Research (ICMR)-Centre for Cancer Pathology, New Delhi, India

**Keywords:** pediatric tumor microenvironment, therapeutic resistance, immune checkpoint inhibition, ECM, developmental oncology

## Abstract

The tumor microenvironment (TME) in pediatric cancers is profoundly shaped by the unique biological context of childhood development. Unlike adult tumors, pediatric malignancies arise in growing tissues, where evolving immune systems, dynamic stromal elements, and distinct hormonal and microbial influences converge to create highly specialized tumor-supportive niches. This review explores how developmental processes interact with tumor biology to drive immune evasion, therapy resistance, and disease progression. Key mechanisms such as extracellular matrix remodeling, metabolic reprogramming, and epigenetic plasticity are highlighted as critical contributors to treatment failure. Also, the recent advancements in nanomedicine, circulating markers makes it possible for interventions to be more precise and age-informed. The integration of developmental biology along with tumor ecosystem can emphasis the necessity of developing treatment plans that take into account risks and advantages associated with pediatric TME.

## 1 Introduction

The tumor microenvironment (TME) plays a crucial role in shaping pediatric cancer behavior, impacting tumor growth, immune evasion, and resistance to therapy. Unlike adult malignancies, pediatric tumors develop within tissues that are still maturing, resulting in TME characteristics that are strongly influenced by age-specific biological and structural factors (1, 2). Adult cancers tend to have distinct immune microenvironments, which can affect treatment outcomes and therapeutic efficacy in ways that differ from pediatric cases (2). Pediatric tumors often respond more favorably to chemotherapy and radiation, contributing to improved survival rates over time (2).

In pediatric tumors, the extracellular matrix (ECM) provides a structural framework, while loosely organized basement membrane scaffolds, along with a mix of soluble and insoluble signaling molecules such as chemokines and cytokines, create a distinctive biochemical and physical environment that sets these tumors apart from adult cancers (1, 3). Pediatric solid tumors show age-specific incidence patterns, with embryonal tumors predominating in early childhood and bone and soft tissue sarcomas becoming more common during adolescence (4). The ECM in pediatric tumors undergoes continuous remodeling, with fibroblasts playing an active role in shaping its structure (5–7). The interplay of cytokines and chemokines helps establish the distinct characteristics of the pediatric tumor microenvironment (8–10). These structural and molecular differences between pediatric and adult TMEs are summarized in **Table 1**.

**Table 1:** Pediatric versus adult tumor microenvironment: ECM and soluble factors.

Feature	Paediatric TME	Adult TME	References
ECM Composition	Enriched with developmental ECM proteins (laminin, fibronectin, collagen III); dynamic and plastic ECM that’s may enhance drug penetration	Denser fibrotic ECM dominated by collagen I & IV; stiffer matrix influencing cell behavior and limiting therapeutic access	(1, 6, 8)
Basement Membrane	Loosely organized; reflects tissue morphogenesis during growth; highly remodeled in pediatric tumors, potentially facilitating both invasions and drug delivery	Well-defined, stiffened basement membrane contributing to barrier function and therapy resistance	(3, 11)
Soluble Factors (Cytokines)	Elevated VEGF, TGF-β, IGF-1 levels promoting angiogenesis and growth; active developmental signaling pathways	Elevated IL-6, TNF-α, IFN-γ promoting chronic inflammation and immune evasion	(12–14)
Chemokines	Active CXCL12/CXCR4 axis driving migration and angiogenesis; CCL22 recruitment of Tregs for immune suppression	Predominantly CCL2, CCL5, CXCL8 (IL-8) driving immune cell recruitment and supporting fibrosis	(15–17)
Fibroblast Activity	Immature CAFs secreting developmental ECM proteins; lower α-SMA expression compared to adults	Mature, highly activated CAFs promoting dense ECM and resistance to immune infiltration	(1, 3, 8)
Immune Cell Infiltration	Higher proportions of Tregs andM2 macrophages sustained by ECM components; less mature immune system with potential for immune modulation	More heterogeneous immune infiltrates; T cell exhaustion in dense ECM microenvironments	(6, 16)

ECM, Extracellular matrix; CAFs, cancer-associated fibroblasts; Tregs, Regulatory T cell; α-SMA, alpha-smooth muscle actin; VEGF, vascular endothelial growth factor; TGF-β, transforming growth factor-beta; IGF-1, insulin-like growth factor 1; IL-6, interleukin-6; TNF-α, tumor necrosis factor-alpha; IFN-γ, interferon-gamma.

**Figure 1:**
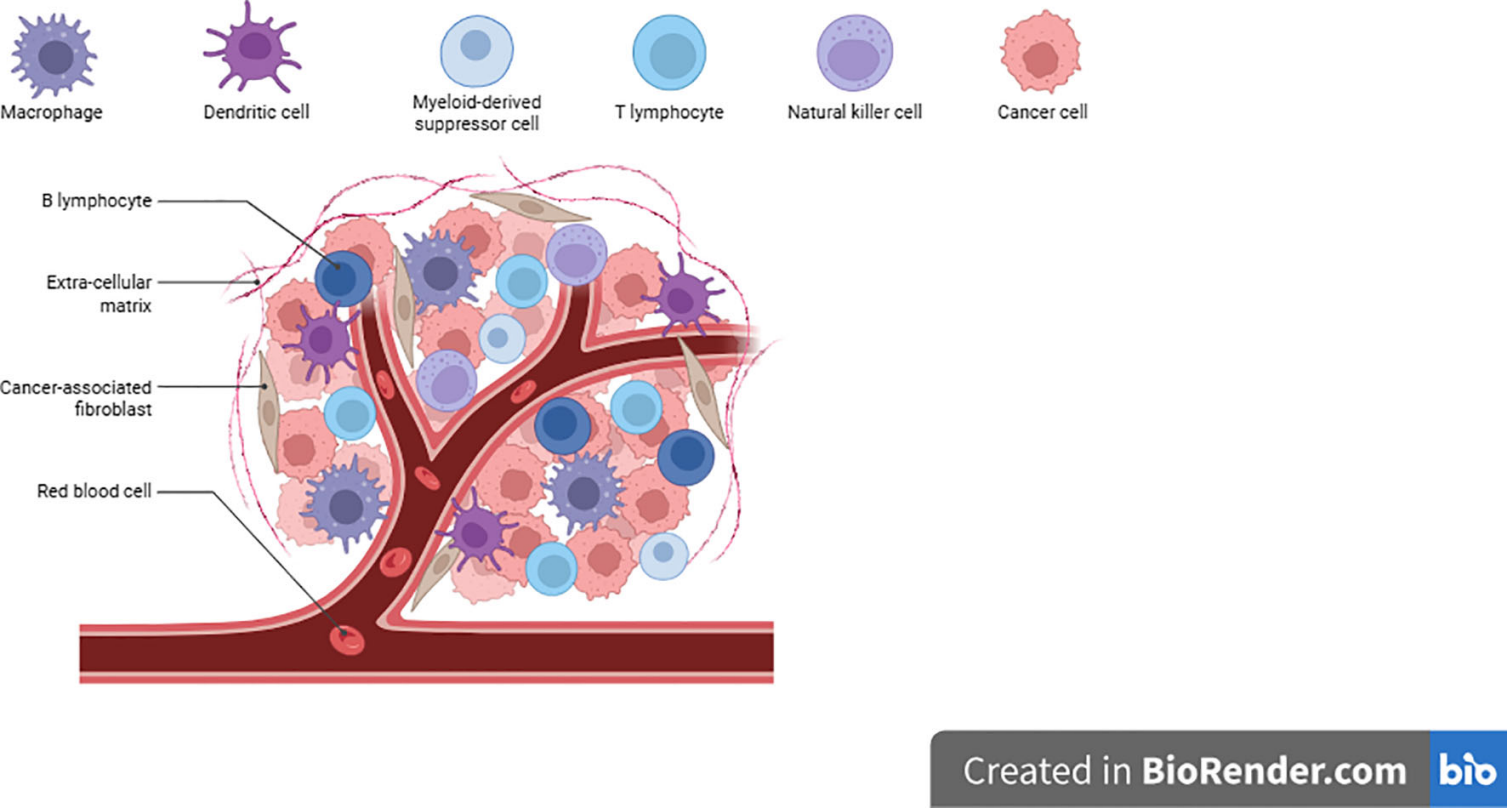
The tumor microenvironment comprises a complex network of tumor cells, immune cells, stromal components like cancer-associated fibroblasts (CAFs), and an extracellular matrix (ECM). These elements form physical and biochemical barriers that hinder immune infiltration and therapy delivery (12).

The cellular components of TME consists of tumor-associated macrophages (TAMs), immune cells, endothelial cells, and cancer-associated fibroblasts (CAFs), as well as non-cellular elements like ECM proteins, lipids, cytokines, and growth factors. These elements not only dictate tumor behavior but also influence responses to therapy (**Figure 1**).

Understanding the pediatric TME requires an integrated exploration of its developmental, systemic, and translational dimensions. Unlike adult tumors, pediatric cancers arise within a dynamically evolving physiological context influenced by growth, hormonal shifts, immune maturation, and microbial colonization. These developmental cues not only shape the biological behavior of tumors but also influence how the TME interacts with therapeutic interventions. Therefore, future research must extend beyond cellular and molecular characterizations to include age-specific hormonal profiles, microbiome signatures, and developmental signaling pathways. Equally important is the translation of these insights into clinical practice through biomarker discovery, patient-derived models, and precision therapies tailored to the pediatric TME. A holistic approach that captures these intersecting layers is critical to improving outcomes for children with solid tumors.

## 2 Overview of tumor microenvironment

### 2.1 Unique characteristics of pediatric TME compared to adults

Pediatric tumors often develop in highly proliferative tissues with structural and vascular differences from adults. The ECM in pediatric tumors is enriched with laminin, fibronectin, and collagen III and serves as a dynamic supporting matrix that fosters tumor growth and immune modulation (1, 6). Unlike the rigid ECM in adults, pediatric ECM is plastic and more susceptible to remodeling. Similarly, basement membrane scaffolds remain immature and loosely organized during tissue morphogenesis, facilitating tumor cell invasion (3, 11).

Non-cellular mediators include both soluble factors (VEGF, TGF-β, IGF-1) and insoluble components like ECM proteins and glycoproteins that establish biochemical gradients influencing tumor behavior (3, 8). The chemokine and cytokine networks also differ between pediatric and adult tumors, which often rely on CXCL12/CXCR4 signaling to drive migration and angiogenesis, whereas adult TMEs exhibit proinflammatory cytokine profiles (IL-6, TNF-α) that support immune evasion (9, 18).

**Figure 2:**
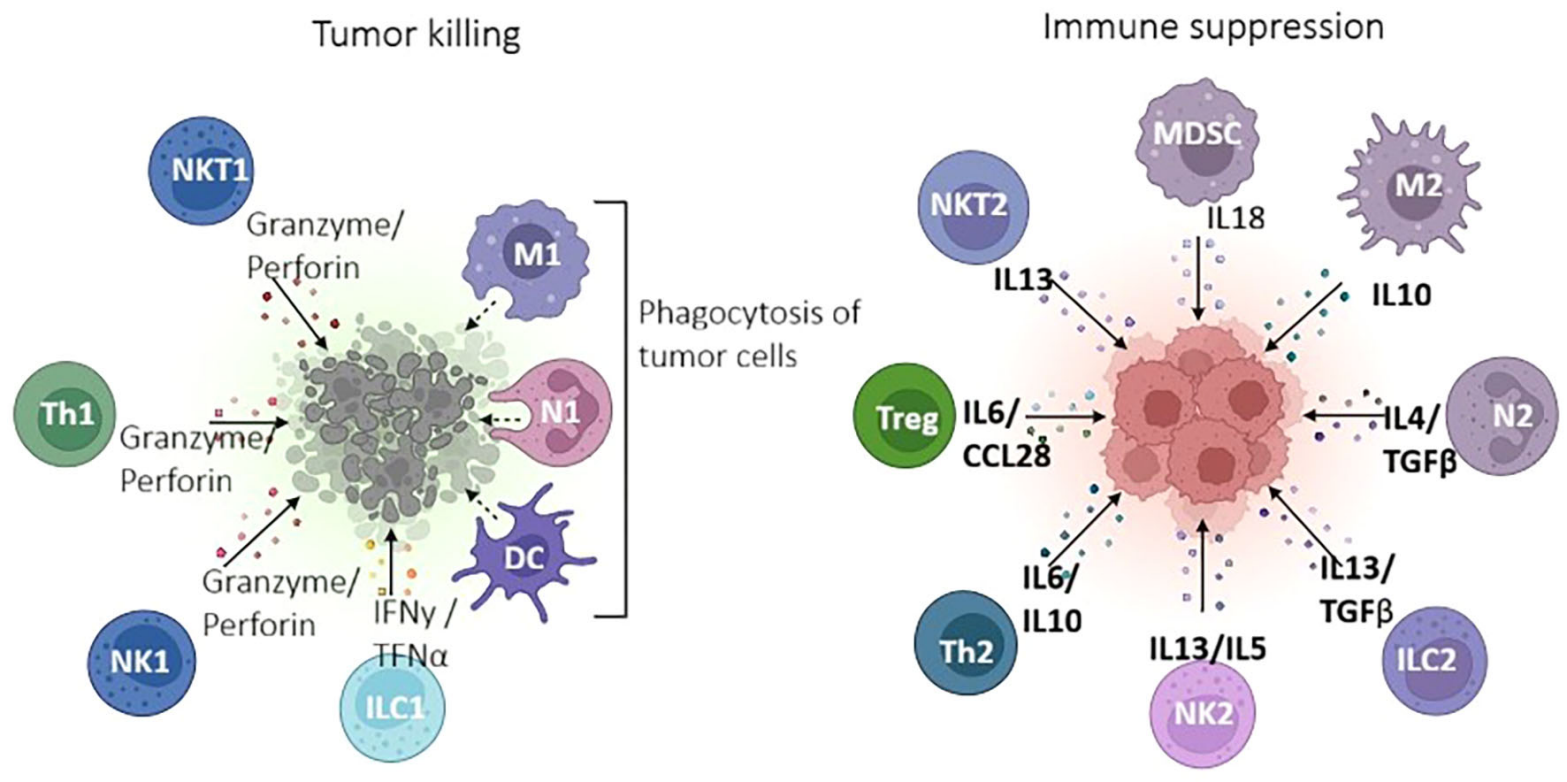
Roles of immune cells in the pediatric tumor microenvironment (TME). Immune effector cells (left) such as NK cells, Th1 cells, and dendritic cells promote tumor cell killing via cytokine release and cytotoxic granules. Conversely, immunosuppressive populations (right) including Tregs, MDSCs, and M2 macrophages suppress anti-tumor immunity through IL-10, TGF-β, and other inhibitory signals, contributing to immune evasion and therapy resistance (Biorender).

The immune contexture differs as well, with pediatric TMEs displaying fewer antigen-presenting cells and a predominance of immunosuppressive populations like Tregs and M2 macrophages compared to adult tumors (6, 18). These immune cell populations play critical but opposing roles in tumor immunity as illustrated in **Figure 2**,

### 2.2 Age specific development in pediatric TME

The pediatric tumor microenvironment (TME) undergoes developmental changes that significantly influence tumor progression, immune modulation, and therapeutic responsiveness. As children mature, the evolving immune and stromal landscape within the TME reflects both intrinsic developmental processes and tumor-induced reprogramming. These changes underscore the need for age-adjusted therapeutic approaches in pediatric oncology.

Age-specific immune responses and the gradual maturation of immune cells shape the developmental pathways underlying immune evasion in pediatric tumors. Cancers in children arise within a distinct immunological environment, where the maturation of the immune system interacts with tumor cells in ways that differ markedly from adults. This interaction is defined by evolving immune cell profiles and functional responses that change throughout early childhood. For example, infants possess an immature, hypo-inflammatory immune system, which may limit their ability to mount effective anti-tumor responses (27). Koutsogiannaki (2023) demonstrated that innate immune responses dominate during early development and that leukocyte functions and transcriptomic profiles undergo significant changes from infancy to school age. Consistent with this, many pediatric tumors exhibit rapid growth and minimal leukocyte infiltration; the immune infiltrate is often enriched in M2-like macrophages and naïve-like T cells, both of which are less capable of initiating robust anti-tumor activity (28). This distinctive immunological landscape suggests that therapeutic strategies aimed at immune modulation particularly those promoting a pro-inflammatory environment could enhance treatment efficacy in children (29). Furthermore, the dynamic nature of the paediatric immune system may offer unique opportunities to develop innovative immunotherapeutic strategies that leverage its developmental characteristics. Recent studies show that immune cell activity in pediatric solid tumors changes as children age. Longitudinal transcriptomic profiling demonstrates a progressive decline in antitumor immune gene expression and an increase in tolerogenic and immunosuppressive signatures as children age (30). This shift reflects a transition from an immunologically active (“hot”) to an immune-depleted or tolerogenic (“cold”) TME, which may contribute to reduced responsiveness to immunotherapies and increased tumor resilience over time.

Beyond immune components, fibroblasts within the TME also undergo age-related changes that influence tumor behavior. Studies in adult pancreatic cancer models have shown that aged fibroblasts promote enhanced tumor cell proliferation, invasiveness, and ECM remodeling, suggesting that stromal aging can exacerbate malignancy (3, 6). Although direct data in pediatric tumors are limited, similar fibroblast-driven modifications to the TME are likely to occur, potentially altering tumor growth dynamics and immune accessibility as children grow.

### 2.3 Organ- specific development in pediatric TME

Organ-specific age-specific biological environments within the TME play a critical role in shaping tumorigenesis, cellular plasticity, and therapeutic response, particularly in pediatric cancers. Far from being a passive structural backdrop, the TME actively regulates tumor phenotype through complex interactions between resident cells and environmental cues. In pediatric gliomas, for example, distinct regional microenvironments give rise to intra- and inter-tumoral heterogeneity, highlighting how local context can drive divergent tumor behaviors (4). In pancreatic cancer, regulatory T cells (Tregs) can either support or suppress tumor growth depending on how they interact with local stromal and immune cells, showing how flexible immune responses can be in different tissue environments (13). These differences are also influenced by normal developmental processes, such as branching morphogenesis, which shapes organ architecture through signaling cues and ECM remodeling. While these processes are crucial for healthy tissue formation, they can reappear in tumors, influencing shape, growth, and progression (31). Studies comparing organ development have uncovered shared regulatory patterns that drive organ-specific adaptations, highlighting how local context can determine tumor behavior (32). Even though tissue-specific traits are important, finding pathways that are shared across organs could reveal therapeutic targets with wider relevance (33). Understanding these organ-specific TMEs is especially critical in children, where ongoing development and immature tissues add extra complexity to tumor biology.

In pediatric kidney tumors, such as Wilms tumor, the TME is strongly shaped by kidney-specific developmental processes. Kidney development relies on carefully coordinated interactions between epithelial and mesenchymal cells, guiding nephron formation and vascular patterning processes that tumors can take advantage of (34). The pathways such as WT1, WNT, and IGF2 are often dysregulated in tumor and the surrounding stroma (31). Early childhood is a critical period because kidney growth and function closely follow age, making the pediatric renal TME highly sensitive to developmental cues (4). Environmental factors, like nutrition or toxin exposure, can further affect normal development and tumor susceptibility. To interpret tumor-stroma interactions and for designing therapies that are tailored to young patients in pediatric nephro-oncology it is important to understand the age-dependent environment.

### 2.4 Hormonal influences on the pediatric TME

Hormonal signaling plays a multifaceted and developmentally sensitive role in shaping the pediatric TME, particularly in relation to growth and neurodevelopment. Central to this process is the growth hormone (GH)/insulin-like growth factor 1 (IGF-1) axis, which is essential for postnatal skeletal growth and tissue development. Disruption of this axis, as demonstrated in Tmem263 knockout mice, results in severe dwarfism and skeletal dysplasia due to its regulation of GH receptor expression and IGF-1 levels (35). These alterations have systemic implications, including on stromal remodeling and vascular development key TME processes.

In pediatric patients with growth hormone deficiency (GHD), neuroimaging has revealed reduced gray matter volume and disrupted white matter microstructure, correlating with cognitive and emotional impairments (36). Hormonal imbalances during puberty, especially fluctuations in steroid hormones like estrogen and testosterone, may further modulate neuroimmune circuits and influence the immune composition of the TME (37). Additionally, the growing concern over endocrine-disrupting chemicals emphasizes that environmental factors may exacerbate or mimic hormonal effects, further complicating the developmental trajectory of pediatric TMEs (9). These hormone-driven mechanisms underscore the need for precision in age- and sex-specific treatment approaches in pediatric oncology.

### 2.5 Microbiome interactions with the pediatric TME

The paediatric TME is profoundly shaped by interactions with the microbiome, which modulates immune function, infection susceptibility, and therapeutic efficacy. In early childhood, the developing microbiota plays a vital role in immune education and inflammation regulation—factors that are particularly relevant for children undergoing chemotherapy or immunotherapy. Dysbiosis, or microbial imbalance, has been associated with increased infection risk, compromised mucosal immunity, and greater treatment-related toxicity in paediatric cancer patients (38, 39).

Microbiome composition can significantly influence treatment response across multiple body sites (40). Specific microbial signatures have been linked to differential outcomes in chemotherapy, immunotherapy, and antibiotic exposure, impacting drug metabolism, immune surveillance, and local TME dynamics (38, 39). In hematopoietic stem cell transplant (HSCT), balancing the microbiome can improve tolerance and reduce risk of graft-versus-host disease (GVHD) (38). For instance, *Akkermansia muciniphila* promotes anti-tumor immunity by activating T lymphocytes and macrophages, demonstrating direct microbiome-immune crosstalk within the TME (41).

Beyond the gut, the oral and skin microbiomes contribute to both local mucosal and systemic immune responses. In atopic dermatitis (AD), patients exhibit lower microbial diversity across oral, skin, and gut compartments compared to healthy children, with increased colonization by *Staphylococcus aureus* that aggravates inflammation and weakens barrier function (42, 43). Early probiotic use has been associated with reduced AD occurrence, highlighting the microbiome’s role in immune tolerance during early life (43). The oral microbiome influences not only dental health but also systemic inflammatory states (44, 45), while the skin microbiome evolves rapidly during infancy, shaped by delivery mode, antibiotic use, and environmental exposures (46). Bacterial genera such as *Cutibacterium* and *Staphylococcus* play dual roles—either maintaining homeostasis or promoting inflammatory conditions that may alter the local TME in rare paediatric cutaneous malignancies.

Emerging therapeutic strategies such as fecal microbiota transplantation (FMT) and probiotic supplementation show promise in restoring microbial balance and improving overall treatment tolerance and efficacy (47, 48). Manipulating the microbiome to reduce systemic inflammation or enhance immunotherapy outcomes represents a novel frontier in paediatric oncology. However, the dynamic nature of the paediatric microbiome, particularly in immunocompromised children undergoing treatment, poses risks including infection and treatment-related complications due to microbial imbalances. Despite potential benefits, it is crucial to consider downsides such as increased inflammation or development of antibiotic resistance. Further research is needed to establish safe and effective microbiome-based approaches tailored to paediatric oncology contexts.

Differences in nutrient availability and metabolic programming between paediatric and adult TMEs also reflect developmental distinctions. Children’s TMEs are influenced by rapid growth demands and higher nutrient needs relative to body weight, affecting both composition and metabolism of the microenvironment (49). Maternal nutrition during gestation can epigenetically program offspring tissues, influencing nutrient availability and TME properties in childhood cancers. Paediatric tumors often rely on altered metabolic pathways, such as aerobic glycolysis (Warburg effect), supporting rapid proliferation despite lower energy yield (50). These early-life exposures may induce lasting epigenetic changes affecting cancer risk and therapeutic response, whereas adult TMEs are shaped by cumulative environmental exposures, chronic inflammation, and lifestyle factors necessitating age-specific therapeutic approaches.

### 2.6 Sexual dimorphism in pediatric TME

Sex-specific differences critically influence the paediatric tumor microenvironment (TME), shaping immune composition, stromal behavior, and therapy response particularly during puberty when hormonal and metabolic shifts occur. Estrogen and androgen signaling modulate T cell activation, cytokine profiles, and stromal remodeling, while X-linked immune genes further contribute to sexual dimorphism in immune regulation (51, 52). Estrogen enhances antibody and Th2 responses (52), whereas sex-dependent variations in CD8+, Treg, and myeloid cell populations alter tumor immunity (51, 52).

Despite well-documented sex-based disparities in immunotherapy outcomes among adults, paediatric oncology trials rarely include sex as a biological variable. The growth hormone (GH)/insulin-like growth factor 1 (IGF-1) axis shows sexual dimorphism with implications for postnatal growth and tissue development (35), while sex-specific drug metabolism via cytochrome P450 enzymes affects efficacy and toxicity. Incorporating sex-stratified analyses in future paediatric TME studies is essential to enable sex-informed precision therapies that improve treatment outcomes while reducing developmental toxicity (51, 52).

Key Points: Pediatric TME Overview.

- Pediatric TME differs fundamentally from adult TME due to ongoing tissue development and immune system maturation
- The ECM in pediatric tumors is more flexible and easily remodeled, enriched with developmental proteins compared to the rigid, dense matrix in adult tumors
- Children’s immune systems evolve throughout childhood, creating age-dependent vulnerability windows for immunotherapy
- Developmental signals uniquely shape pediatric TME in ways not seen in adults
- Organ-specific developmental programs influence tumor behaviors
- Understanding these age-specific feature is critical for developing effective pediatric cancer treatment

## 3 Mechanisms of therapy resistance driven by the TME

Therapy resistance in paediatric cancers arise from the intersection of developmental biology and tumor evolution, creating age-specific vulnerabilities and challenges distinct from adult malignancies. Paediatric tumors frequently hijack embryonic signaling cascades that remain active or easily reactivatable during childhood. Unlike adult tumors, which usually result from accumulated genetic and cellular damage, paediatric tumors arise in rapidly developing tissues with unique metabolic needs that influence growth and survival strategies (53, 54).

Paediatric tumors exhibit biological traits unique to their developmental context that affect immune evasion and treatment response. These tumors frequently display decreased leukocyte infiltration, with a preponderance of M2-like macrophages and naïve-like T cells that impair antitumor immunity (29). Myeloid-derived suppressor cells (MDSCs) are essential for reducing immunological responses; in medulloblastoma, for instance, MDSCs reduce T cell activity, facilitating immune escape (33). Many paediatric tumors feature a developmental maturation block that might reduce the efficacy of conventional treatments, calling for innovative therapeutic approaches specific to their biology (53). The response of paediatric and adult malignancies to immunotherapies and targeted treatments is also influenced by differences in active signaling pathways (55). Developing more efficient, age-appropriate treatment plans for paediatric malignancies require understanding these age-dependent variations.

The TME creates multiple interconnected barriers to effective treatment through physical, molecular, and immunological mechanisms. Below, we detail the major resistance mechanisms organized by category.

### 3.1 Physical and structural barriers

The extracellular matrix (ECM) and associated physical features create substantial obstacles to drug delivery and immune cell infiltration. The dense ECM, enriched with fibronectin and collagen, physically prevents drug penetration into tumors (22, 56). Beyond acting as a barrier, ECM components interact with cancer cells to trigger survival signals that increase drug resistance (22). Dynamic ECM remodeling, particularly pronounced in paediatric tumors where fibroblasts actively shape stromal architecture (5–7), continuously adapts to therapeutic pressure.

Hypoxia represents a critical physical characteristic driving multiple resistance mechanisms. Low oxygen conditions typical of solid tumors activate hypoxia-inducible factor (HIF) pathways, which enhance cancer cell adaptability, promote epithelial-mesenchymal transition (EMT), and facilitate immune evasion (22). Hypoxia-driven metabolic reprogramming allows tumors to survive in nutrient-depleted environments while simultaneously excluding immune effector cells.

Drug efflux mechanisms further limit therapeutic efficacy. Many cancer cells produce large amounts of ATP-binding cassette (ABC) transporters, which actively expel drugs from cells, lowering intracellular drug concentrations and reducing treatment effectiveness (56). Cancer stem cells possess particularly robust drug efflux capabilities, helping tumors survive therapy and develop resistance (56). The TME’s acidic conditions further contribute to resistance by altering pH partitioning at cell membranes, leading to extracellular accumulation of chemotherapeutic agents that would otherwise passively diffuse into cancer cells (57).

### 3.2 Immunological barriers and immune evasion

Immune evasion mechanisms within the TME significantly impairs therapeutic responses. While some tumors respond well to immune checkpoint inhibitors, others do not, highlighting intrinsic differences in resistance development patterns. In paediatric solid tumors, high levels of TGF-β, IL-10, and VEGF contribute to immune suppression and T cell exclusion. Immune checkpoint pathways including PD-1/PD-L1 and CTLA-4, while well-validated targets in adult cancers, show limited efficacy in paediatric contexts, with response rates consistently below 5% in sarcomas (58).

New immunotherapies are attempting to reshape the tumor environment to improve treatment efficacy in paediatric cancers. Targeting stromal TME components with FAK inhibitors or TGF-β blockers, often in combination with immune checkpoint inhibitors (ICIs), has shown promise in improving T cell infiltration. This mechanistic interplay between the TME and immune modulation is critical for advancing effective immunotherapies in paediatric oncology. The predominance of immunosuppressive populations regulatory T cells (Tregs), M2 macrophages, and MDSCs creates a tolerogenic microenvironment that actively suppresses anti-tumor immunity through secretion of IL-10, TGF-β, and other inhibitory signals (6, 18, 59).

### 3.3 Metabolic reprogramming and adaptation

Metabolic reprogramming driven by the TME represents a key contributor to therapy resistance (60). Cancer cells adapt to metabolic stress induced by treatments through several interconnected mechanisms. The shift to aerobic glycolysis (Warburg effect) involves increased glucose uptake and lactate production to support rapid proliferation and survival under therapeutic stress (61). Enhanced lipid metabolism in both primary and metastatic tumors provide essential energy and structural components, reinforcing resistance mechanisms. Alterations in amino acid metabolism sustain protein synthesis and energy production under therapeutic pressure (61).

The TME induces stress response secretory programs (SRSPs), where non-cancerous stromal cells secrete cytokines and growth factors that protect tumor cells during treatment. Increased inflammatory signaling within the TME strengthens cancer cell survival and fosters acquired resistance (17). Cancer-associated fibroblasts (CAFs) release signaling molecules that alter tumor cell treatment responses, creating a protective niche that enhances therapeutic resistance (8, 15).

### 3.4 Cellular plasticity and epigenetic reprogramming

Cancer cells exhibit lineage plasticity—the ability to alter their phenotype under therapeutic stress allowing them to evade treatment by adopting less differentiated or alternative cell states (62, 63). This plasticity enables tumors to bypass targeted therapies by shifting to phenotypes not recognized by the therapeutic agent. Lineage plasticity is closely linked to metabolic reprogramming and is often accompanied by epigenetic changes, where metabolites directly influence chromatin modifications, thereby promoting tumor progression and therapeutic resistance (63). Stromal cells, particularly CAFs, further promote adaptive cellular programs such as epithelial-mesenchymal transition (EMT), enhancing tumor invasiveness and therapy resistance (56).

Epigenetic reprogramming plays a crucial role in treatment resistance, particularly in aggressive paediatric and adult malignancies such as gliomas. These modifications do not alter DNA sequence but modify gene expression patterns, allowing cancer cells to adjust quickly to treatment and immune pressures. Histone acetylation and DNA methylation changes can silence tumor-suppressor genes and activate cancer-promoting pathways, helping tumor cells grow, survive, and evade the immune system (41, 64). These modifications also shape the immune environment around tumors, creating suppressive settings enriched with regulatory T cells (Tregs) and myeloid-derived suppressor cells (MDSCs) (41, 65). Consequently, fewer cytotoxic T cells can enter and function properly, lowering immunotherapy success rates.

Drugs targeting epigenetic changes show promise. Entinostat, an HDAC inhibitor, helps the immune system identify tumor cells more easily and improves immunotherapy efficacy (65). Preclinical research demonstrates that combining epigenetic drugs with immune checkpoint inhibitors or cell-based therapies can boost antitumor effects, reduce tumor size, and extend survival. However, tumors remain adaptable and can develop new resistance mechanisms even during epigenetic therapy. Further research is needed to determine how long these treatments remain effective and how the tumor environment changes over time.

### 3.5 Integrated resistance networks

While targeting metabolic pathways represents a promising therapeutic approach, it may inadvertently promote further plasticity or new resistance mechanisms, highlighting the complexity of this strategy. The interconnected nature of these resistance mechanisms—physical barriers, immune suppression, metabolic adaptation, and epigenetic flexibility—creates robust, multi-layered defense systems that challenge single-agent therapies. Despite formidable TME-driven resistance mechanisms, emerging strategies such as combination therapies and precision medicine approaches are being developed to counteract these barriers and improve therapeutic outcomes (66).

### 3.6 Temporal evolution of resistance

Resistance in pediatric cancers evolves dynamically as the TME remodels under therapeutic pressure. Early chemotherapy induces tumor cell death and damage-associated molecular pattern (DAMP) release, triggering inflammatory immune infiltration that creates a transient window for immune activation. However, surviving tumor cells simultaneously upregulate stress response programs, while cancer-associated fibroblasts (CAFs) secrete protective factors that shield residual disease from subsequent treatment cycles. Between treatment cycles, vascular normalization occurs approximately 1–3 days after anti-VEGF therapy, potentially enhancing drug delivery if chemotherapy is optimally timed. However, prolonged anti-angiogenic pressure triggers compensatory angiogenesis through alternative pathways (FGF, PDGF), explaining why initial responses cannot be sustained. The pediatric TME, with inherently higher vascular plasticity due to ongoing development, may undergo more rapid remodeling than adult tumors. Longitudinal immune profiling reveals progressive shifts from activated to exhausted phenotypes (67). In pediatric gliomas, this transition from immunologically “hot” to “cold” TME occurs over 6–12 months (30), suggesting immunotherapy may be most effective when introduced early rather than reserved for relapsed disease. Epigenetic reprogramming accelerates during treatment, with each cycle selecting for increasingly adaptable clones. Matched diagnosis-relapse pairs in sarcomas confirm accumulated modifications affecting DNA repair, drug efflux, and immune evasion pathways. The kinetics of resistance evolution are influenced by treatment schedules. Studies in pathogenic bacteria demonstrate that temporal variation in drug exposure—fluctuating between high and low concentrations—can slow resistance evolution compared to constant intermediate exposure (68). Fluctuating selection pressures prevent consistent directional selection, instead favoring phenotypic plasticity over fixed resistance mutations (68). Translating these principles to cancer therapy, intermittent dosing schedules may limit selection for stable resistance mechanisms. Given rapid developmental remodeling in pediatric tissues, real-time monitoring via circulating biomarkers, liquid biopsies, or imaging is vital to capture TME evolution and optimize treatment timing. Future clinical trials should incorporate planned biopsies at multiple timepoints or employ circulating markers to identify optimal temporal windows for introducing TME-targeted interventions. Understanding not just what mechanisms drive resistance, but when they emerge during treatment, will enable rational sequencing of therapies to preempt rather than merely respond to resistance evolution.

Key Points: TME-Driven Therapy Resistance (**Table 2**).

**Table 2:** Key tumor microenvironment components in pediatric solid tumors.

Tumor type	Cellular components	Non-cellular components	TME-driven features	References
Neuroblastoma	Schwann cells, neuroblastic cells, TAMs, MSCs	ECM stiffness, IL-6, SDF-1, IGF-1/2, RANKL, acidic pH	Immune evasion, bone metastasis, N-Myc modulation, drug resistance	(14, 18–20)
Rhabdomyosarcoma	CAFs, endothelial cells, MDSCs, Tregs, TAMs	TGF-β, VEGF, fibronectin, ECM remodeling enzymes	Angiogenesis, immune suppression, invasion, migration	(6, 8, 9, 21)
Medulloblastoma	Microglia, astrocytes, endothelial cells, Tregs	SHH pathway, ECM proteins, hypoxia-induced factors	Blood-brain barrier challenges, immune evasion, therapy resistance	(2, 11, 18, 19)
Ewing Sarcoma	Osteoblasts, MSCs, TAMs	CXCL12/SDF-1, hypoxia, ECM stiffness, IGF pathway molecules	Bone niche colonization, ECM-mediated signaling, therapy evasion	(3, 8, 18, 22)
Wilms Tumor	Fibroblasts, endothelial cells, macrophages	TGF-β, VEGF, ECM collagen, IGF-II	Stromal modulation, proliferation signals, angiogenesis	(3, 6, 7, 21)
DSRCT	Desmoplastic fibroblasts, TAMs, endothelial cells	Dense ECM, VEGF, PDGF, IL-6, TGF-β	Desmoplasia, immune evasion, chemoresistance	(8, 9, 22, 23)
Lymphomas (HL & NHL)	B-cells, T-cells, TAMs, dendritic cells, fibroblasts	IL-10, TGF-β, PD-L1, ECM components	Immune suppression, immune checkpoint activation, stromal support	(14, 24–26)

This table summarizes the major cellular and non-cellular components of the tumor microenvironment in common pediatric solid tumors, including neuroblastoma, rhabdomyosarcoma, medulloblastoma, Ewing sarcoma, and Wilms tumor. It highlights how each tumor type’s unique TME contributes to disease progression, therapy resistance, and metastasis.

- Paediatric tumors have unique resistance mechanisms driven by developmental biology
- Physical barriers: dense ECM, drug efflux pumps, and poor drug penetration
- Immune evasion: M2 macrophages, MDSCs, and Tregs suppress anti-tumor immunity in paediatric TME
- Metabolic adaptations: Warburg effect (aerobic glycolysis), altered lipid/amino acid metabolism sustain tumor survival under therapy
- Epigenetic reprogramming: Histone modifications and DNA methylation allow rapid adaptation to treatment pressure
- Lineage plasticity: Tumor cells can change identity to evade therapy
- Combination approaches targeting multiple TME components show more promise than single-agent strategies
- Temporal evolution: resistance mechanisms shift dynamically; optimizing treatment timing through real-time monitoring is key to overcoming resistance.

## 4 Current therapeutic interventions targeting the pediatric TME

Current therapeutic strategies targeting the TME in pediatric cancers focus on disrupting key pathways that promote tumor growth, immune evasion, and therapy resistance. Among these, anti-angiogenic therapies have emerged as a particularly promising approach.

### 4.1 Tumor-specific heterogeneity and clinical outcomes in TME-targeted therapy

The pediatric TME exhibits profound heterogeneity across tumor types, directly influencing therapeutic response and necessitating tumor-specific treatment approaches. Understanding these differences is essential for rational treatment selection and predicting clinical outcomes. Current FDA-approved and clinical-stage therapies targeting the pediatric TME are listed in **Table 3**.

**Table 3:** Current FDA-approved or clinical-stage therapies targeting pediatric TME.

Therapeutic class	Agent (s)	Indication/target	Status	Citation
Checkpoint Inhibitor	Pembrolizumab	TMB-high unresectable/metastatic solid tumors	FDA-approved for pediatric use	(70)
CAR T-cell Therapy	Various experimental platforms	Pediatric brain tumors (clinical trials)	Clinical-stage	(42)
Biologics (anti-IL/anti-JAK)	Ritlecitinib, Secukinumab, Dupilumab	Pediatric dermatological/inflammatory disorders	FDA-approved/Phase 3 trials	(27)
Genomic-Guided Therapies	N/A (target-dependent)	Pediatric solid tumors with actionable mutations	Clinical trials, limited approvals	(41)
Targeted Small Molecules	Pediatric-specific molecular agents	Tailored pediatric TME modulation	In development/clinical-stage	(34)

#### 4.1.1 Clinical translation: landmark successes and instructive failures

##### 4.1.1.1 Neuroblastoma: proof-of-concept for TME-targeted immunotherapy

The Children’s Oncology Group ANBL0032 trial established anti-GD2 immunotherapy as the first successful TME-targeted approach in paediatric solid tumors, providing proof-of-concept that targeting tumor-specific antigens can bypass TME-mediated immunosuppression (64, 69). This randomized phase III trial enrolled 226 high-risk neuroblastoma patients who had achieved complete or very good partial response after intensive multimodal therapy. The combination of dinutuximab (anti-GD2 monoclonal antibody) with GM-CSF, interleukin-2, and isotretinoin significantly improved outcomes compared to standard maintenance therapy. Two-year event-free survival increased from 46% in the control arm to 66% in the immunotherapy arm (p=0.01), while 2-year overall survival improved from 78% to 86% (64).

The mechanism involves activation of natural killer cells and macrophages through antibody-dependent cellular cytotoxicity (ADCC), effectively overcoming the dense desmoplastic stroma and immunosuppressive cytokine milieu (TGF-β, IL-6) characteristic of high-risk neuroblastoma (8, 64). This trial led to FDA approval of dinutuximab in 2015, marking the first TME-modulating immunotherapy approved for paediatric cancer (64). The success demonstrates that even in challenging TME contexts—dense stroma, elevated immunosuppressive cytokines, poor T-cell infiltration, targeted approaches exploiting tumor-specific antigens can achieve meaningful clinical benefit (8, 18).

##### 4.1.1.2 Ewing sarcoma: when TME architecture determines therapeutic failure

In stark contrast, Ewing sarcoma exemplifies immune-cold TME where architectural features predict therapeutic failure. These tumors present uniformly minimal lymphocyte infiltration, low mutational burden, and dense ECM with active CXCL12/CXCR4 signaling that physically excludes immune cells (3, 8, 22). The extracellular matrix creates insurmountable physical barriers preventing immune access (1). Clinical trial data demonstrate consistently dismal response rates to single-agent checkpoint inhibitors, with response rates below 5% across multiple studies (58). This striking contrast 66% event-free survival improvement in neuroblastoma versus <5% in Ewing sarcoma demonstrates that TME architecture, not tumor genetics alone, determines immunotherapy potential. The failure in Ewing sarcoma underscores that immune infiltration capacity matters more than tumor mutational burden for predicting immunotherapy response (18, 58).

##### 4.1.1.3 Rhabdomyosarcoma: age-dependent TME shifts and treatment response

Rhabdomyosarcoma demonstrates how age-dependent TME evolution influences treatment efficacy, necessitating developmental stage-specific strategies rather than histology-based approaches alone. The embryonal subtype, predominant in younger children, demonstrates high VEGF expression with immature cancer-associated fibroblasts secreting developmental ECM proteins (1, 6, 8). This developmental context confers enhanced responsiveness to metronomic anti-angiogenic approaches, as sustained low-dose therapy effectively targets the immature vasculature characteristic of younger patients’ tumors (70). Conversely, the alveolar subtype, more common in older children and adolescents, displays mature, activated CAFs with different stromal composition requiring targeting of PAX3-FOXO1 fusion alongside TME components (1, 7, 21). This age-dependent shift suggests that clinical trial design should incorporate age stratification based on TME maturity, not merely histologic classification.

##### 4.1.1.4 Wilms tumor: when developmental TME enhances treatment

Wilms tumor represents a unique scenario where developmental TME characteristics enhance rather than impede chemotherapy efficacy. The tumor microenvironment closely resembles normal nephrogenesis, with cellular and stromal components mimicking kidney development patterns (31, 71). This developmental similarity results in rapidly dividing, highly chemo sensitive cells, contributing to >90% overall survival with standard chemotherapy protocols (vincristine, dactinomycin, with or without doxorubicin) (71). The success reflects how developmental TME features can create therapeutic vulnerability rather than resistance.

However, anaplastic Wilms tumor variants with increased cancer-associated fibroblast activity and elevated TGF-β demonstrate markedly inferior outcomes, revealing how TME shifts from treatment-permissive to treatment-restrictive microenvironment can occur even within the same tumor type (3). This underscores the critical importance of TME profiling for risk stratification and treatment selection (1, 3).

##### 4.1.1.5 Checkpoint inhibitors: the TMB paradox in paediatric cancers

Pembrolizumab received FDA accelerated approval for paediatric patients with unresectable or metastatic solid tumors exhibiting high tumor mutational burden (TMB-high), based on a 29% overall response rate in this molecularly selected population (65). However, applicability to most paediatric solid tumors remains limited, as the majority are characterized by low TMB (<10 mutations/mega base) and immune-cold microenvironments with minimal lymphocytic infiltration (2, 18). These disappointing results underscore that TMB alone inadequately predicts response in paediatric cancers, where physical barriers (dense ECM, immune cell exclusion) and cellular composition (predominance of immunosuppressive populations) play dominant roles (1, 18, 58) (**Table 4**).

**Table 4:** Clinical outcomes by TME-Targeted approach in pediatric tumors.

Tumor type	TME features	Therapy	Trial/study	Clinical outcome	Ref
Neuroblastoma (high-risk)	Dense stroma, high TGF- β/IL-6, immune cold	Anti-GD2 (dinutuximab) + GM-CSF + IL-2 + isotretinoin	COG ANBL0032 (n=226) NCT00026312	66% 2-yr EFS; 86% 2-yr OS	(8, 18, 20, 73)
Ewing Sarcoma	Minimal immune infiltration, dense ECM, active CXCL12/CXCR4	Checkpoint inhibitors (pembrolizumab, nivolumab)	KEYNOTE-051/other trials	<5% ORR	(3, 8, 18, 22, 58)
Rhabdomyosarcoma (embryonal)	High VEGF, immature CAFs, age-dependent	Metronomic chemotherapy ± anti-angiogenic		Enhanced response in younger patients	(1, 8, 66)
Wilms Tumor (favorable histology)	Developmental TME mimicking nephrogenesis	Standard chemotherapy (vincristine, dactinomycin ± doxorubicin)	COG AREN0532/0533	>90% 4-yr OS	(1, 7, 31, 74)
Wilms Tumor (anaplastic)	Increased CAF activity, elevated TGF-β, therapy-resistant stroma	Standard chemotherapy		Inferior outcomes; requires TME-targeted strategies	(3)

This table summarizes how tumor microenvironment (TME) characteristics influence therapeutic response and prognosis across major pediatric solid tumors, highlighting differences in stromal composition, immune contexture, and treatment efficacy.

##### 4.1.1.6 Anti-angiogenic strategies: context-dependent efficacy

Anti-angiogenic approaches targeting VEGF signaling demonstrate variable efficacy depending on tumor type and treatment regimen (32, 70). Metronomic dosing—continuous administration of low-dose chemotherapy with anti-angiogenic effects—shows promising results in specific paediatric contexts. A study of recurrent paediatric ependymoma utilizing metronomic 5-drug anti-angiogenic therapy achieved 78% two-year progression-free survival, suggesting that sustained anti-angiogenic pressure may be more effective than intermittent high-dose approaches in the developmentally active paediatric TME (72). This continuous low-dose strategy may prevent compensatory activation of alternative angiogenic pathways that often limit efficacy of intermittent anti-VEGF therapy (32, 70).

However, anti-VEGF monoclonal antibody therapy (bevacizumab) in recurrent paediatric CNS tumors provide only modest benefit as monotherapy, with progression-free survival improvements typically limited to 1–3 months (32, 70). This limited efficacy likely reflects rapid tumor adaptation through activation of alternative angiogenic pathways (FGF, PDGF, angiopoietins) within the dynamic paediatric TME (32). These findings suggest that combination approaches simultaneously targeting multiple angiogenic pathways or combining anti-angiogenic therapy with immune modulation may be necessary for durable responses (70).

#### 4.1.2 Principles for paediatric TME-targeted therapy

Current clinical evidence reveals several critical insights. First, TME characteristics particularly immune infiltration capacity and stromal architecture determine therapeutic vulnerability independent of tumor genetics (18, 58). Second, combination approaches targeting multiple TME components show superior efficacy compared to single-agent strategies (58, 70). Third, age-specific TME features necessitate paediatric-informed trial designs with age stratification (29). Fourth, biomarkers developed in adult cancers (PD-L1 expression, TMB) poorly predict paediatric response, highlighting urgent need for paediatric-specific predictive biomarkers (58, 65). Ongoing research incorporating TME-specific endpoints immune infiltration patterns, stromal remodeling markers, angiogenic profiles alongside traditional efficacy measures will be essential to advance the field (18, 58, 70). Understanding not just whether but why certain paediatric TMEs respond to specific therapies will enable rational treatment selection and combination strategy design (1, 8, 64).

#### 4.1.3 Case example: targeting the TME in pediatric solid tumor

Recent advancements in extracellular matrix (ECM)-targeting therapies have demonstrated significant potential in overcoming therapy resistance in pediatric solid tumors. Joshi (2020) study examined how modifying the extracellular matrix (ECM) can improve treatment outcomes in pediatric cancers. The study showed that targeting the stromal components of the tumor microenvironment (TME) can make therapies more effective. For example, the drug galunisertib, a TGF-β inhibitor, was found to block tumor-promoting signals from fibroblasts in the TME. When used together with immune checkpoint inhibitors, galunisertib boosted immune activity and promoted tumor shrinkage, showing a strong synergistic effect.

Beyond drugs like galunisertib, biomimetic nanocarriers are emerging as a promising tool to deliver treatments more precisely. These nanocarriers mimic the structure and function of the ECM, allowing them to target tumors accurately and overcome drug delivery barriers in dense pediatric tumors. Such strategies could also be applied to other pediatric solid tumors, such as neuroblastoma and rhabdomyosarcoma, by tailoring ECM-modulating therapies to the specific features of each tumor type.

The challenge now is actually using these approaches in real patient’s pediatric tumors are incredibly complex and vary widely. Future research needs to focus on improving the specificity of ECM-targeting methods, reducing side effects, and enhancing their effectiveness across different pediatric cancers Such strategies could be expanded to other pediatric solid tumors, including neuroblastoma and rhabdomyosarcoma, by designing ECM-modulating therapies tailored to their unique microenvironmental compositions. **Figure 1** provides a visual summary of therapeutic approaches targeting the neuroblastoma TME, showing strategies that modify the ECM, immune cells, and tumor blood vessels to improve treatment responses (31).

### 4.2 Challenges in translating TME-targeted therapies to pediatric patients

Developmental toxicity represents the foremost concern. Anti-angiogenic agents target VEGF signaling essential for growth plate ossification, dental development, and organ maturation (32, 58). Preclinical studies demonstrate impaired bone growth and delayed sexual maturation in juvenile animals, necessitating dose reductions that compromise efficacy. Checkpoint inhibitors cause higher rates of severe immune-related adverse events in children than adults: thyroiditis (18% vs 8%), type 1 diabetes (5% vs 1%), hypophysitis (12% vs 3%) (70, 75). These differences occur because developing immune systems have narrower therapeutic windows between anti-tumor activity and autoimmunity. These endocrine toxicities may require lifelong hormone replacement, an unacceptable burden for cured paediatric patients. Long-term cardiovascular, fertility, and neurocognitive impacts remain inadequately studied.

Pharmacokinetic differences across paediatric age groups complicate dosing. Infants have immature hepatic enzymes causing prolonged drug half-lives, children aged 2–12 require higher mg/kg dosing due to faster metabolism, and adolescents experience unpredictable drug distribution during puberty’s rapid body composition changes (76). Over 80% of TME-targeted therapies lack paediatric pharmacokinetic data, and body surface area extrapolation from adults fails to account for developmental TME differences.

Small patient populations create insurmountable statistical barriers. Most pediatric solid tumors affect fewer than 200 new US patients annually (19). Ethical constraints prohibit placebo controls when effective chemotherapy exists, necessitating demonstration of added benefit without increased toxicity. Age stratification further fragments samples, while standard endpoints fail to capture 30-50-year developmental impacts.

Economic and regulatory barriers limit investment. Small market size provides insufficient incentive for pediatric-specific formulations despite high development costs. FDA pediatric exclusivity provisions have proven inadequate. Many agents are used off-label based solely on adult data, but insurance often denies coverage for off-label pediatric use. Adult TME biomarkers (PD-L1 expression, tumor mutational burden) poorly predict pediatric response, yet developing pediatric-specific companion diagnostics requires separate regulatory approval (75).

Biological complexity of the rapidly evolving pediatric TME poses unique challenges. The microenvironment changes over months as children grow, creating temporal dynamics that complicate treatment timing (77). Preclinical models inadequately recapitulate pediatric TME: adult mice lack developmental context, juvenile models exist for few tumor types, patient-derived xenografts lose pediatric characteristics in adult mice, and organoids lack immune/stromal components (78, 79). Limited data on normal pediatric tissue TME across age spectrum creates uncertainty about developmental impact of TME-targeted interventions.

What this really means is that we can’t just give children smaller doses of adult cancer drugs. Paediatric TME-targeted therapy needs its own approach one that accounts for how children’s bodies develop and grow, while also thinking about quality of life for kids who survive into adulthood.

### 4.3 Biomarker-guided therapy selection

Effective TME-targeted therapy in paediatric oncology depends on biomarker-guided precision strategies rather than empirical selection. Immune infiltration metrics (e.g., CD8+/FOXP3+ ratios, spatial exclusion) and composite immunoscores predict immunotherapy response, though pediatric validation remains incomplete (80). Stromal and angiogenic signatures such as CAF markers, collagen profiles, and the Xerna™ TME Panel differentiate subtypes with distinct responses to FAK or TGF-β blockade (25, 80).

Integrative multi-omics models and AI-based platforms (e.g., Lunit SCOPE IO) improve predictive power (16), while adaptive and Bayesian trial designs address small paediatric cohorts and imperfect biomarker accuracy (81). Paediatric tumors’ low mutational burden, fusion-driven biology, and age-dependent immunity demand tailored biomarker interpretation (80). Standardized, minimally invasive monitoring using ctDNA, CTCs, and extracellular vesicles (78, 79) can support real-time, biomarker-informed decisions, transforming paediatric oncology into a precision-guided therapeutic framework (80, 81).

Key Points: TME-Targeted Therapies: Clinical Evidence.

Successes:

- Neuroblastoma + anti-GD2: 66% vs 46% EFS (ANBL0032, n=226, FDA-approved)
- Wilms Tumor: >90% cure with standard chemotherapy (developmental enhances response)

Challenges:

- Ewing sarcoma + checkpoint inhibitors: <5% response (uniform immune exclusion)
- Most paediatric tumors: low TMB and immune-cold TME limit immunotherapy
- Bevacizumab monotherapy: Only 1–3-month PFS benefit

Ongoing Research:

- FAK inhibitors + checkpoint inhibitors for ECM remodeling
- TGF- β blockers to reduce stromal immunosuppression
- Nanomedicine for improved drug delivery through TME barriers
- Microbiome modulation to enhance immunotherapy

## 5 Future directions: TME as a therapeutic target

Future therapeutic strategies targeting the TME, particularly through nanomedicine and combination therapies, hold considerable promise in improving pediatric cancer outcomes. Researchers are actively developing advanced nanomedicines designed to enhance drug delivery, maximize therapeutic efficacy, and minimize systemic toxicity (66, 82). Innovations such as responsive nanomedicines, which specifically respond to TME characteristics, and theragnostic platforms that integrate diagnosis and therapy are paving the way for more personalized treatment approaches (82).

Combination strategies, including the integration of nanomedicines with immunotherapies and antiangiogenic agents, have demonstrated synergistic effects in preclinical models, significantly improving treatment responses. Additionally, multimodal approaches such as combining photodynamic therapy with conventional chemotherapy offer further potential to enhance therapeutic efficacy through complementary mechanisms (66).

Studies show we are still in the early days of actually using nanomedicine and AI diagnostics for pediatric cancer patients. Challenges related to scalable manufacturing, regulatory approval, and affordability must be addressed before widespread clinical adoption can occur. Moreover, the heterogeneity of the TME adds complexity to treatment responses, necessitating highly tailored therapeutic strategies.

Circulating tumor DNA (ctDNA) has emerged as a highly promising biomarker for monitoring the TME and evaluating therapeutic responses across a range of pediatric and adult malignancies. ctDNA comprises fragmented DNA released by tumor cells into the bloodstream and enables non-invasive, real-time assessment of genetic and epigenetic alterations associated with cancer. In pediatric and adult contexts alike, ctDNA shows potential for early diagnosis, treatment monitoring, and detection of therapy resistance or recurrence. For example, ctDNA profiling has enabled early detection of hepatocellular carcinoma (HCC) through identification of tumor-specific mutations before radiologic changes occur (75), and in undifferentiated pleomorphic sarcoma, ctDNA levels were shown to rise with recurrence and fall post-chemotherapy, correlating with complete pathological response (77). In colorectal cancer, ctDNA-based assays have successfully identified actionable gene mutations and tracked resistance development, with changes in ctDNA mutation burden reflecting treatment efficacy and disease progression (78). Despite these advantages, challenges remain including sequencing sensitivity, managing high-throughput data, and accurately correlating ctDNA concentration with tumor burden. Nevertheless, ctDNA represents a transformative tool in TME analysis, offering non-invasive and dynamic insight into pediatric cancer evolution, therapeutic efficacy, and relapse surveillance.

Circulating immune cell profiling within the TME offers critical insights into tumor biology and holds considerable promise for guiding immunotherapeutic strategies in pediatric cancers. Emerging evidence has shown that immune cell infiltration patterns vary widely between tumor types, contributing to tumor progression, immune evasion, and treatment response. For instance, in malignant pleural mesothelioma, distinct immune landscapes have been mapped using automated nine-color multiplex immunofluorescence, revealing spatial interactions between immune subsets and tumor cells (79). Similarly, in pancreatic cancer, immune cell infiltration (ICI) scores have been identified as useful prognostic biomarkers and lower ICI scores are linked to better clinical outcomes (83). Research through single-cell RNA sequencing has found the diversity and complexity of immune cells within TME and this allows for more precise understanding of immune cell functions in various tumors (26). In head and neck squamous cell carcinoma, clinical studies have shown that higher levels of certain immune cells are linked to better responses to immune checkpoint inhibitors. These findings highlight the value of integrating immune profiling into clinical studies to match patients with the most effective therapies. The lack of standardized methods and difficulties in interpreting large, complex datasets is still a major hindrance in making immune profiling a routine part of pediatric cancer care, larger studies and standardized approaches are needed.

TME-derived biomarkers are becoming powerful tools to personalize cancer treatment by matching therapies to the specific features of each tumor. Artificial intelligence (AI) and machine learning are playing a growing role in this effort by helping quickly identify and validate predictive biomarkers. These biomarkers can guide the use of immunotherapies and anti-angiogenic treatments, improve their effectiveness while avoid unnecessary treatments.

For example, AI-based models like Lunit SCOPE IO analyze features of the TME, including tumor-infiltrating lymphocytes (TILs), to predict treatment outcomes. In hepatocellular carcinoma (HCC) patients, this analysis has shown strong links with progression-free survival, demonstrating its clinical potential (16). Similarly, the Xerna™ TME Panel, an RNA expression-based classifier, categorizes tumors into distinct TME subtypes and has demonstrated improved predictive accuracy over conventional biomarkers (25).

However, limitations persist. Low clinical trial enrolment rates in pediatric oncology, driven by the rarity of pediatric cancers and ethical considerations surrounding trials involving children, remain major barriers to rapid advancement (84). Additionally, concerns regarding the long-term efficacy of TME-targeted therapies and the risk of inducing resistance highlight the critical need for continued research. Going forward, we need to figure out how to manufacture nano therapies at scale, deal with the fact that every tumor environment is different, and create age-appropriate treatment strategies designed specifically for children’s biology.

## 6 Conclusion

The TME in pediatric cancers plays a pivotal role in driving therapy resistance and disease progression. By influencing immune evasion, promoting angiogenesis, remodeling the extracellular matrix, and modulating metabolic pathways, the TME creates a complex and dynamic environment that challenges conventional treatment approaches. Recent advances in therapeutic strategies, including ECM-targeting agents, anti-angiogenic therapies, immune checkpoint inhibitors, and nanomedicine-based delivery systems, offer new avenues for disrupting these TME-driven resistance mechanisms. This dynamic ECM remodeling and fibroblast activity are particularly pronounced in pediatric tumors.

However, challenges such as tumor heterogeneity, the rarity of pediatric cancers, and the long-term safety of novel interventions remain significant barriers. The next step is understanding pediatric TME at a deeper molecular level, using AI to identify new biomarkers, and designing combination therapies built specifically for pediatric tumors. Understanding and effectively targeting the unique features of the pediatric TME is essential for improving therapeutic outcomes and advancing precision oncology for young patients.
