# Correction: Pediatric tumor microenvironment: developmental dynamics and therapy resistance

**DOI:** 10.3389/fonc.2025.1745674

**Published:** 2025-11-21

**Authors:** Noreen Grace George, Bhavika Rishi, Himanshu Dhanda, Gratial Theres Joseph, Sandeep Kumar Swain, Manpreet Kaur, Neetu Kushwaha, Raj Kamal, Pranay Tanwar, Sufian Zaheer, Shamsuz Zaman, Amitabh Singh, Fouzia Siraj, Aroonima Misra

**Affiliations:** 1 Indian Council of Medical Research-National Institute of Child Health Development Research, New Delhi, India; 2 Manipal Academy of Higher Education (MAHE), Manipal, India; 3 All India Institute of Medical Sciences, New Delhi, India; 4 Vardhman Mahavir Medical college, Safdarjung Hospital, New Delhi, India; 5 Indian Council of Medical Research (ICMR)-Centre for Cancer Pathology, New Delhi, India

**Keywords:** pediatric tumor microenvironment, therapeutic resistance, immune checkpoint inhibition, ECM, developmental oncology

Affiliation “Manipal Academy of Higher Education (MAHE), Manipal, India” was omitted for authors Noreen Grace George, Himanshu Dhanda, Gratial Theres Joseph.

This affiliation has now been added for authors Noreen Grace George, Himanshu Dhanda, Gratial Theres Joseph.

The **Conflict of interest** statement has been corrected to “The authors declare that the research was conducted in the absence of any commercial or financial relationships that could be construed as a potential conflict of interest.”

The original version of this article has been updated.

